# The Role of Medical Language in Changing Public Perceptions of Illness

**DOI:** 10.1371/journal.pone.0003875

**Published:** 2008-12-08

**Authors:** Meredith E. Young, Geoffrey R. Norman, Karin R. Humphreys

**Affiliations:** 1 Department of Psychology, Neuroscience and Behaviour, McMaster University, Hamilton, Ontario, Canada; 2 Department of Clinical Epidemiology and Biostatistics, McMaster University, Hamilton, Ontario, Canada; University of Toronto, Canada

## Abstract

This study was designed to investigate the impact of medical terminology on perceptions of disease. Specifically, we look at the changing public perceptions of newly medicalized disorders with accompanying newly medicalized terms (e.g. impotence has become erectile dysfunction disorder). Does using “medicalese” to label a recently medicalized disorder lead to a change in the perception of that condition? Undergraduate students (n = 52) rated either the medical or lay label for recently medicalized disorders (such as erectile dysfunction disorder vs. impotence) and established medical conditions (such as a myocardial infarction vs. heart attack) for their perceived seriousness, disease representativeness and prevalence. Students considered the medical label of the recently medicalized disease to be more serious (mean = 4.95 (SE = .27) vs. mean = 3.77 (SE = .24) on a ten point scale), more representative of a disease (mean = 2.47 (SE = .09) vs. mean = 1.83 (SE = .09) on a four point scale), and have lower prevalence (mean = 68 (SE = 12.6) vs. mean = 122 (SE = 18.1) out of 1,000) than the same disease described using common language. A similar pattern was not seen in the established medical conditions, even when controlled for severity. This study demonstrates that the use of medical language in communication can induce bias in perception; a simple switch in terminology results in a disease being perceived as more serious, more likely to be a disease, and more likely to be a rare condition. These findings regarding the conceptualization of disease have implications for many areas, including medical communication with the public, advertising, and public policy.

## Introduction

In recent years, the definition of disease and ‘non-disease’ has led to a heated debate in the literature [1]–[10], with much of the rhetoric focused around the ‘medicalization’ of syndromes and disorders on the fringes of the classic definition of disease [2]–[8], [11]–[13]. The use of medical terms to describe such disorders as male pattern baldness (androgenic alopecia), chronic fatigue syndrome (myalgic encephalopathy), and impotence (erectile dysfunction disorder), to mention a few [14], appear to coincide with a trend towards the “medicalization” of society. This trend has been addressed in domains stretching from philosophy [3], [5], [15], to health policy and expenditures [4], to the cultural status of disease [10], to the impact on patient and health care worker views of illness [2], [6], [7], [15], and has lead to much debate regarding the role of advertising in disease mongering [12]–[13], [16]–[18].

This shift towards medical labels for a wide range of diseases, disorders, syndromes and symptoms has created a new kind of medical language. Some medical terminology is a direct derivative of Latin or Greek-like equivalents (e.g. myalgic encephalopathy ‘translates’ literally to ‘aching disease of the brain’ [15]); others are English-based, but are still in a formal/technical register that has connotations of special medical status (e.g. Erectile Dysfunction Disorder (as opposed to the term ‘impotence’)). For the remainder of this paper, both will be referred to as ‘medicalese.’

A critical question here concerns how and why these changes in both terminology and in public perceptions of disorders are taking place. Specifically, to what extent is the public perception driving the terminology, and to what extent is the terminology driving public perception. If the former, it might be that the social criteria for what constitutes a disease are changing, and that society is becoming more accepting of conditions previously considered to be at the fringes of health. This then might make the adoption of more formal medical terminology for these disorders seem more appropriate. Alternatively, are the terminological changes, the renaming of disorders, creating a new understanding of an old illness? Is the creation of new medical terminology validating and accrediting disorders previously considered outside of the traditional biomedical realm? In this case, the original impetus for the terminological changes may come not from changes in general public perception, but from other stakeholders. However, it is important to note that the relationship between the terminological change and public perception may not be simple and unidirectional, and it is possible that terminology and public opinion influence each other. However, for the purposes of this paper, we hope to begin to explore the impact of medical language on disease perception.

A few previous studies have examined perceptions of different diseases across a variety of medical and non-medical populations. Previous research has demonstrated a privileged status of medical terminology in the diagnostic process of medical students [19] and residents [20], where participants assigned significantly more diagnostic weight to a disorder described using medicalese than one described using lay-English equivalents. Campbell *et al.* identified differential treatment of disease synonyms by physicians [1], and Jason *et al.* demonstrated the impact of a medical disease label on medical students' perceptions of severity, prognosis, and cause [15]. These studies have all demonstrated a clear, and seemingly irrational influence of terminology – individuals across different levels of expertise and understanding are treating synonymous disease labels differentially.

In this paper, we will investigate the impact of medical and lay disease labels in both established medical disorders and newly medicalized disorders. We predict that the impact of medicalese should be particularly powerful in the case of newly medicalized conditions, where public perceptions are likely still in flux. If this is the case, this provides evidence for the use of new terminology (e.g. in advertising) leading to a shift in understanding of a particular illness. In contrast, we predict that recognized medical conditions will be considered to have equal disease status, be equally serious, and be equally prevalent regardless of the label used to identify the disorder.

## Methods

### Participants

A total of 52 participants were recruited from an introductory psychology course subject pool. All participants were undergraduate students at McMaster University, and were compensated with experimental course credit. Ethical approval for this study was obtained from the McMaster University Research Ethics Board.

### Study Design

Participants were briefed regarding the procedure of the study, and written informed consent was obtained. Participants were given a survey consisting of 34 different medical disorders, of which 16 will be discussed in this paper (the remainder were part of a larger, ongoing study). These 16 disorders represent two different categories: 8 were chosen due to the increased popular use of a medicalese label within the last 10 years (e.g. erectile dysfunction, chronic fatigue syndrome, androgenic alopecia), the remaining 8 are established medical disorders that have both lay and medical terminology that have been in popular use for more than 10 years (e.g. heart attack, hypertension, celiac disease). The remaining data were collected for an on-going study that examined several other medical linguistic phenomena.

Participants were asked to make three different judgments for each disorder: seriousness (on a scale of 1 to 10, where 1 was not very serious and 10 was very serious), prevalence (in a sample of 1,000 of other students their age, how many people were likely to have the disorder in the next year), and disease representativeness (how likely the disorder was representative of a disease where 1 = definitely not a disease, 2 = probably not a disease, 3 = probably a disease, 4 = definitely a disease). Participants were randomly assigned to one of two groups, where each participant was asked to rate either the medicalese or lay label for a disorder. Participant group and order of presentation of disease labels was determined using a random number generator. Language of presentation was counterbalanced across groups, meaning that no participant saw both the lay and medicalese terminology for the same disorder.

In order to investigate the role of immediately present information on judgments of seriousness, disease-like status and prevalence, participants were randomly assigned to either a Low Information or High Information condition. In the Low Information condition, participants were asked to make judgments based only on the name of the disorder. In the High Information condition, participants were asked to make judgments based on the name, symptoms, indications of prevalence, fatality, and transmission vector of the disorder. Note that the prevalence information provided was not prevalence statistics for the peer group that participants were asked to evaluate, rather population statistics were provided within the disease description. An example of the Informational conditions is shown in Table 1.

**Table 1:** Sample of low and high informational conditions.

Informational Condition	Example
Low Information	Chronic Dandruff
High Information	Chronic Dandruff: Chronic dandruff is characterized by itchy flaking skin that appears on the scalp or eyebrows, or around the hairline, ears or nose. Flakes of skin can range from small and white to large, greasy and yellow. 4% of people in Canada will have chronic dandruff in their lifetimes.

### Materials

Disease labels chosen for this study were disorders that have recently been medicalized, including those that have no current biological understanding of causation, or those that have had a lifestyle drug released for its treatment within the last 10 years. Participants also rated 8 established medical disorders for contrast purposes. In order to validate the category assignment of these disorders, a Lexis Nexis search was conducted sampling from 1997 and 2007 for frequency of use of both the medial and lay labels for each disorder in popular news media (defined as print media publicly available in North America). When examining the recently medicalized disorders in print media, we see a six-fold increase in the ratio of medicalese to lay labeling, where we see only a 1.2 fold increase in the same ratio for recognized medical disorders. A list of the disorders used in this study, and their subcategory assignment are found in Table 2.

**Table 2:** List of lay and medicalese terminology used and associated subcategory assignment.

Subcategory	Lay Terminology	Medicalese Terminology
Recognized Medical Condition	High Blood Pressure	Hypertension
	Gall Bladder disease	Cholecystitis
	Celiac Disease	Gluten-induced enteropathy
	Stroke	Cerebrovascular Accident
	Lou Gherig's Disease	Amyotrophic lateral sclerosis
	Heart Attack	Myocardial Infarction
	Cushing's Disease	Hypercortisolism
	Sore Throat	Pharyngitis
Recently medicalized Disorders	Impotence	Erectile Dysfunction Disorder
	Chronic Fatigue Syndrome	Myalgic Encephalomyelitis
	Male Pattern Baldness	Androgenic Alopecia
	Dandruff	Seborrheic Dermatitis
	Chronic Heartburn	Gastroesophageal Reflux Disease
	Excessive Hair Growth	Hypertrichosis
	Excessive Sweating	Hyperhidrosis
	Skin Tags	Acrochordon

We would like to make clear that by making the abovementioned distinctions, we do not intend to disregard the suffering of individuals with any of the disorders used in this study, nor do we claim that individuals with these disorders do not have a disease. We make this distinction solely in order to examine the effects of medical terminology on the perceptions of healthy, lay individuals within the undergraduate community, and these examples are chosen as cases where perceptions of the condition may be currently in the process of changing.

### Analysis

A repeated measures ANOVA was conducted with type of disorder (established vs. recently medicalized disorders) and label language (medicalese/lay label) as the two within-subjects factors for ratings of seriousness, disease representativeness and prevalence. Between-subjects factors included information condition.

## Results

### Overall analysis

An overall analysis was performed to evaluate the effect of language (medicalese vs. lay) and the impact of the type of disorder rated (newly medicalized vs. established medical conditions) on ratings of seriousness, disease representativeness and prevalence. For ratings of seriousness, a main effect of type of disorder was found, with participants rating the established medical conditions as more serious than the recently medicalized conditions (mean established medical conditions = 6.79, SE = .226, mean newly medicalized conditions = 4.39, SE = .178, *F* (1,49) = 169.2, *p*<0.001). No main effect for language of presentation (medicalese vs. lay) was found (*F* (1,49) = 2.57, *p* = .12), but a significant interaction of language of presentation and type of disorder was found (*F* (1,49) = 43.81, *p*<0.001).

For ratings of disease representativeness, participants considered the medicalese label to be more representative of a disease than the lay label (mean for medicalese label = 2.50, SE = .07, mean for the lay label = 2.25, SE = .08, *F* (1,49) = 9.87, *p*<0.005). A main effect for type of disorder was also found, with participants rating the established medical conditions to be more representative of a disease (mean established medical conditions = 2.60, SE = .08, mean newly medicalized conditions = 2.15, SE = .07, *F* (1,49) = 41.07, *p*<0.001). Additionally, a significant interaction between type of disorder and language of presentation was found (*F* (1,49) = 56.41, *p*<0.001).

For estimates of prevalence, participants considered the medicalese label to be more prevalent than the lay label (mean for medicalese label = 74.9, SE = 12.7, mean for the lay label = 102.6, SE = 14.9, *F* (1,49) = 13.93, *p*<0.001). No main effect for type of disorder was found (*F* (1,49) = .998, *p* = 0.32). A significant interaction between type of disorder and language of presentation was found (*F* (1,49) = 13.93, *p*<0.001).

The presence of significant interaction between disease type and medical language within each of the measures: seriousness, disease representativeness and prevalence, indicates that the role of medicalese language may differ between the recently medicalized and established medical conditions. Separate analyses of newly recognized and established medical disorders is warranted in order to establish the specific role of medical language in both of these categories of disorders.

### Recently medicalized disorders

**Figure 1:**
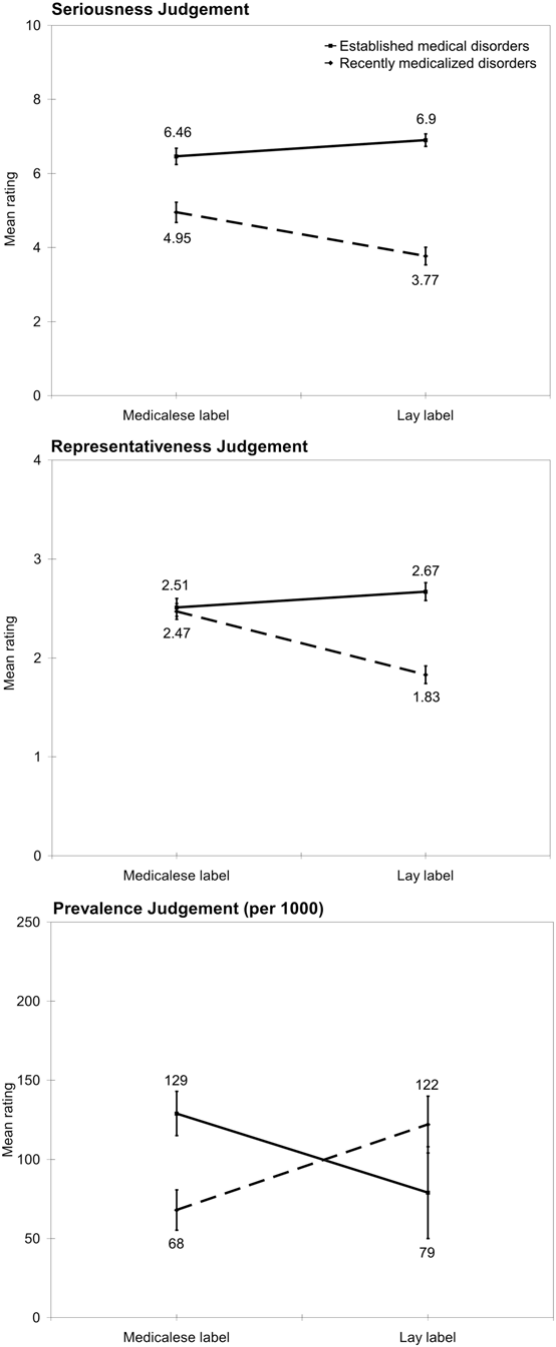
Mean judgments of seriousness, disease-representativeness, and prevalence for newly medicalized and established medical conditions for both medicalese and lay disease labels. Error bars represent standard error of the mean.

Participants reported that the medicalese label for the recently medicalized disorders was more serious (4.95 (SE = .27) vs. 3.77 (SE = .24) on a ten point scale, *F* (1,49) = 28.9, *p*<0.001, see Figure 1, top panel), had higher disease representativeness (mean for medicalese label = 2.47, SE = .08, mean for lay label = 1.83, SE = .09 on a four point scale, *F* (1,49) = 45.1, *p*<0.001, see Figure 1, center panel), and was less prevalent (mean for medicalese label = 68 individuals, SE = 12.7, mean for lay label = 122, SE = 18.0 individuals out of 1000, *F* (1,49) = 13.67, *p*<0.001, see Figure 1, bottom panel) than its lay terminology counterpart, collapsed across information conditions.

When the data were analyzed by information condition (high vs. low), the recently medicalized diseases showed a main effect for ratings of seriousness (mean for low information condition = 5.25, SE = .22, mean for high information condition = 3.47, SE = .21 on a ten point scale, *F* (1,49) = 22.9, *p*<0.001) and disease-like status (mean for low information condition = 2.34, SE = .10, mean for high information condition = 1.96, SE = .10 on a four point scale, *F* (1,49) = 7.3, *p*<0.01), but not for prevalence. The interaction between informational condition and medical or lay language was not significant for seriousness, disease representativeness, or prevalence. This lack of a significant interaction indicates that the medicalese label being considered to be more serious and have higher ratings of disease representativeness is not significantly altered by the presence of additional information.

### Established medical disorders

For the established medical disorders, there was no significant difference between the ratings assigned to the medicalese and lay name for disease representativeness (mean for medicalese label = 2.51, SE = .89, mean for lay label = 2.67, SE = .09 on a four point scale, *F* (1,49) = 3.33, *p* = .08, see Figure 1, center panel). Participants reported that the lay version of the established medical disorders was more serious (mean for medicalese label = 6.46, SE = .22, mean for lay label = 6.90, SE = .17 on a ten point scale, *F* (1, 49) = 10.99, *p*<0.005, see Figure 1, top panel) and was less prevalent (mean for lay label = 79 individuals, SE = 16.7, mean for medicalese label = 129, SE = 14.0, individuals out of 1,000, *F* (1,49) = 12.31, *p*<0.05, see Figure 1, bottom panel).

When the data were analyzed by information condition (high vs. low), the recently medicalized diseases showed a main effect of condition for ratings of seriousness (low information condition = 7.14, high information condition = 6.39 on a ten point scale, *F* (1,49) = 5.06, *p*<0.05), but not for disease representativeness, nor prevalence. The interaction between informational condition and medical or lay language was not significant for seriousness, disease representativeness, or prevalence for these established medical disorders.

### Post-hoc analyses

One clear difference between the recently medicalized and the established medical disorders is the range of severity. Given that it is difficult to consider male pattern baldness and a heart attack as comparable in severity, it is possible that the difference between medicalese and lay labels is only apparent in disorders that are not considered to be very serious. Perhaps by examining established medical disorders we are observing an impact of severity, rather than an impact of medicalized language. To address this difference, and to further our understanding of the impact of language on perceptions of illness, post hoc analyses on a subset of established medical disorders were conducted.

We examined only established medical disorders that were considered to be as, or less, severe than recently medicalized disorders. A criterion of a mean severity rating of 6.9 was set (the highest mean rating of a recently medicalized disorder); as a result sore throat, Cushing's disease, celiac disease, and gall bladder disease were included in this secondary analysis.

A repeated measures ANOVA was conducted on the restricted list of established medical disorders, in the same manner described above. In this restricted sample of established medical disorders, there was a significant difference in estimates of prevalence (mean for medicalese label = 105 individuals, mean for lay label = 201 individuals out of 1,000, *F* (1,49) = 12.31, *p*<0.005), but no significant difference for ratings of seriousness or disease representativeness. This subgroup analysis indicates that the difference in ratings of severity, disease representativeness and prevalence observed in the newly medicalized conditions is not due to the difference in severity levels between the recognized medical disorders and recently medicalized disorders.

## Discussion

This study demonstrates that a medical label for a recently medicalized disorder results in perceptions of increased severity, increased disease representativeness and lower prevalence compared to the same disorder presented in its synonymous, lay label. This effect is relatively stable across disorders, and remains even when a full description of the disorder is provided. This positive relationship between perceived severity and disease representativeness, which together show an inverse relationship with estimates of prevalence, is consistent with previous findings [19]–[20]. However, this classic medicalese pattern of results is unique to recently medicalized disorders, as this is not seen in established medical disorders, even when severity is equated across the two groups of disorders in post-hoc analyses. If any observations are to be made regarding the established medical conditions, it is that the lay label is seen as more serious than the medicalese, indicating a pattern opposite to that previously seen in the literature [19].

This paper set out to evaluate the role medicalized language is playing in the increased medicalization of disorders previously considered to be at the fringes of health. The data presented here show increased perceptions of seriousness, disease representativeness, and decreased estimates of prevalence for the medicalese labels when applied to recently medicalized disorders. We therefore suggest that the application of medical labels for recently medicalized disorders is functioning to change our understanding of that illness, which may contribute to a shift towards the medicalization of society. This finding also adds to a growing body of literature demonstrating differential treatment of synonymous terms [21].

However, there is also the feeling among some [3]–[6] that a trend towards labeling of medical conditions could result in a perception that the prevalence of these conditions is on the rise - i.e. if it makes it into the public media, it must represent a serious [22], or common, problem. Instead, the current study indicates that the change in nomenclature of a condition is more likely to result in a decrease in perceived prevalence, rather than an increase. The pattern of results found in this study are consistent with previous literature demonstrating that conditions that are rated to be more serious, are also likely to be rated as less prevalent [19].

This study has some limitations. The current design of this study allows us only to infer the existence of a causal relationship – the differential treatment of medical and lay terminology suggests that the shift in language is creating a shift in perception. These data do support the observation that medical language appears to have a ‘privileged status’[20], and lend support to the possible role of advertising [3], [5]–[6] in the medicalization shift. However, further research, perhaps longitudinally, will be able to address the specific nature of institution of medical terminology. Additionally, our study population was limited to undergraduate students, however, previous research has indicated that estimates of risk increase with age [23]. Furthermore, this study indicates a strong role of medical language on perceptions of illness, however this study is unable to identify the specific characteristics of medical language that may be driving this effect. This is a clear direction for future research.

The pattern of results found in this study has obvious implications for communication. If a patient is informed that she has gastroesophageal reflux disease, rather than chronic heartburn, she could consider herself to be more ill, to have a disorder that is more representative of a disease, and more rare (from the lower estimations of prevalence). When a medicalese disease label is used, individuals believe that they suffer from a rare disease, and literature suggests that individuals who believe they have a rarer disorder report greater emotional upset [24] **.** The current investigation does not include patients [25], but suggests an influence of medical language in communication.

Differential understanding of lay and medicalese terminology, and the subsequent effects on lay perceptions of disease seriousness, representativeness and prevalence should be taken into consideration during medical communication with the public. The role of medical language can impact public perception of illness in such domains as advertising and press releases, and has larger implications for the controversy surrounding the medicalization of the disorders along the fringes of health. Additionally, future directions of research include investigating the role of medical language in a patient's decision to seek care and the evaluation of not only medical information drawn from the internet, but decisions to seek treatment, how quickly, and whether to comply with that treatment. This kind of patient decision making, or self-triage [26], has obvious implications for health care and the dissemination of health related information.
